# Functional screening of a human saliva metagenomic DNA reveal novel resistance genes against sodium hypochlorite and chlorhexidine

**DOI:** 10.1186/s12903-021-02000-5

**Published:** 2021-12-09

**Authors:** Johannes Wigand, Supathep Tansirichaiya, Endre Winje, Mohammed Al-Haroni

**Affiliations:** 1 Department of Clinical Dentistry, Faculty of Health Sciences, UiT the Arctic University of Norway, 9037 Tromsø, Norway; 2 Centre for New Antimicrobial Strategies, UiT the Arctic University of Norway, Tromsø, Norway; 3 Department of Microbiology, Faculty of Medicine Siriraj Hospital, Mahidol University, Bangkok, Thailand

**Keywords:** Antimicrobial resistance, Functional metagenomics, Oral metagenomic DNA, Dentistry, Chlorhexidine resistance, Sodium hypochlorite resistance

## Abstract

**Objective:**

Many sections of the health care system are facing a major challenge making infectious disease problematic to treat; antimicrobial resistance (AMR). Identification and surveillance of the resistome have been highlighted as one of the strategies to overcome the problem. This study aimed to screen for AMR genes in an oral microbiota, a complex microbial system continuously exposed to antimicrobial agents commonly used in dental practice.

**Materials and methods:**

As a significant part of the oral microbiome cannot be conventionally cultured, a functional metagenomic approach was chosen. The human oral metagenomic DNA was extracted from saliva samples collected from 50 healthy volunteers in Norway. The oral metagenomic library was then constructed by ligating partially digested oral metagenome into pSMART BAC vector and introducing into *Escherichia coli*. The library was screened against antimicrobials in dental practices. All resistant clones were selected and analyzed.

**Results:**

Screening of the oral metagenomic library against different antimicrobials detected multiple clones with resistance against chlorhexidine, triclosan, erythromycin, tetracycline, and sodium hypochlorite. Bioinformatic analysis revealed both already known resistance genes, including *msr*, *mef(A), tetAB(46)*, and *fabK*, and genes that were not previously described to confer resistance, including *recA* and *accB* conferring resistance to sodium hypochlorite and chlorhexidine, respectively.

**Conclusion:**

Multiple clones conferring resistance to antimicrobials commonly used in dental practices were detected, containing known and novel resistant genes by functional-based metagenomics. There is a need for more studies to increase our knowledge in the field.

**Supplementary Information:**

The online version contains supplementary material available at 10.1186/s12903-021-02000-5.

## Introduction

Antimicrobial agents have saved uncountable numbers of lives for decades since the discovery of Penicillin; however, with a worldwide increase of antimicrobial resistance, infectious diseases currently have become more challenging to be treated. All uses of antimicrobials apply selective pressure to bacteria to evolve and develop antimicrobial resistance [1–4]. Discovery of resistance genes recovered from ancient samples showed that they were significantly similar to the modern resistance variants, suggesting antimicrobial resistance as an old natural phenomenon [5–7], but have recently become a problem possibly due to the selective pressures that accelerated the spreading of resistance genes through horizontal gene transfer [8–10]. Identification and surveillance of the resistome are, therefore, essential in the battle against antimicrobial resistance, as they will improve our understanding of resistance genes in each setting which can be used to design effective strategies to limit the spreading between organisms and environments [11–13].

The human oral cavity is a complex microbial system [14, 15], housing a selection of bacteria with more than 700 bacterial species [16–18]. It consists of several small ecosystems with unique environments such as keratinized and non-keratinized mucosa, the tongue, saliva, tonsils, teeth, and subgingival pockets together making up the oral microbiome [16, 19]. The species in the oral microbiome vary, from facultative aerobes to strict anaerobes. They are continuously exposed to antimicrobial agents from external products such as oral hygiene products as toothpaste, mouth rinse, agents used in dental treatment and food, and is therefore likely to develop antimicrobial resistance. Relevant examples of antimicrobials used in dental practices and dental hygiene products are chlorhexidine used in antimicrobial mouth rinses post-operative of surgical procedures [20], and for gingivitis and periodontitis patients who are unable to maintain adequate mechanical hygiene [21], sodium hypochlorite used as an irrigation agent during root canal treatment [22], sodium benzoate used in various toothpastes, cetyltrimethylammonium bromide (CTAB) found in throat lozenges and topical gels and conventional antibiotics for patients with risk factors pre-operative of surgical procedures. Studies have shown that the oral microbiome contains resistance genes against various antimicrobials agents such as β-lactams, tetracycline, tigecycline, amoxicillin, gentamicin, CTAB, erythromycin and cetylpyridinium chloride [1, 23–28].

Of the more than 700 oral bacterial species, one-third of them are not cultured in the laboratory yet due to difficult and unknown proper conditions for growth [29, 30], which has created challenges for characterizing the resistome in the oral microbiome. Functional metagenomics is a culture-independent approach, which relies on phenotypes of resistance genes, rather than the sequences of the resistance genes as in PCR and microarray [31–33]. It is, therefore, a method with the potential to discover completely novel resistance genes [34–37], without culturing bacteria. It involves cloning of metagenomic DNA into a vector, introducing into a surrogate bacterial host, and screening for clones of phenotypes of interest, such as resistance traits. Several novel resistance genes were identified from the oral metagenome through a functional metagenomic approach, such as tetracycline resistance gene *tet*(37), tigecycline resistance gene *tet*AB(60) and quaternary ammonium compounds (QACs) resistance gene *galE* [1, 23, 24].

In this study, we aimed to detect novel antimicrobial resistance genes from the human oral microbiome through a functional metagenomic approach. A human oral metagenomic library obtained from 50 healthy volunteers in Norway was constructed and used to screen against antimicrobials that are commonly in contact with oral bacteria. We have identified multiple resistance clones against triclosan, CTAB, sodium hypochlorite, chlorhexidine, and erythromycin.

## Methods

### Study participants and collection of saliva samples

Ethical approval was obtained from Regional Committees for Medical and Health Research Ethics (Project number 2018/1373/REK nord). Saliva samples were collected between October and November 2018 from 50 healthy volunteers visiting the University Dental Clinic at UiT The Arctic University of Norway who were invited to participate in the study. All participants gave their written consent to participate in the study. The following criteria were used for participation: no history of antibiotic use in the last three months prior to saliva sampling, no history of regular medication, nor chronic diseases. All volunteers gave their written consent to participate in the study. The volunteers were asked not to drink, eat or brush their teeth within an hour before the collection. A paraffin gum was used to stimulate saliva secretion during collection, and 2 mL of stimulated saliva was collected from each participant into a Saliva DNA Collection and Preservation Kit (Norgen Biotek Corp, Ontario, Canada). All samples were anonymized and stored at room temperature.

### Extraction of oral metagenomic DNA and construction of the oral metagenomic library

Saliva metagenomic DNA was extracted from each sample by mixing 750 μl of each saliva in the preservation tube with 750 μl phosphate-buffered saline (PBS) buffer. This mixture was centrifuged for 10 min at 15,700×*g*. The supernatant was discarded and resuspended in 125 μl PBS and 25 μl MetaPolyzyme (Sigma-Aldrich, Norway). The samples were incubated at 35 °C for 4 h. The DNA samples were then extracted with QIAcube (Qiagen, Norway), following the protocol from QIAamp**®** DNA Mini QIAcube Kit.

For the construction of the oral metagenomic library, 10 μl of extracted DNA was aliquoted from each of the 50 samples, making up 500 μl. The pooled metagenomic DNA was partially digested for 2, 3, and 4 min at 37 °C with HindIII restriction enzyme to serve us large DNA fragments. The digested product was run on an agarose gel electrophoresis, and DNA fragments with a size of more than 1000 bp were extracted by using QIAgen gel extraction kit (Qiagen). The pSMART BAC HindIII vector (7.6 kb) was fully digested and dephosphorylated by using HindIII restriction enzyme and calf intestinal alkaline phosphatase (CIAP) enzyme (NEB, UK) at 37 °C for 60 min. Afterwards, the partially digested oral metagenome was ligated into a pre-digested pSMART BAC vector by using Anza T4 DNA Ligase Master Mix (ThermoFisher, Norway) and incubated for 16 h at 4 °C. The ligation product was desalted in an agarose cone. The desalted ligation product (2 µl) was then mixed with 20 μl BAC-Optimized Replicator (BacRep) *Escherichia coli* Electrocompetent cells (Lucigen, USA), and transferred to a pre-chilled 0.1 cm electroporation cuvette (Bio-Rad, Norway). The mixture was electroporated with the following settings: 1.8 kV, 25 μF, 200 Ω (MicroPulser Electroporator, Bio-Rad, Norway). A pre-warmed recovery medium (950 µl) (Lucigen, USA) was immediately added to the cells and incubated at 37 °C with shaking for 1 h, before plating 100 μl on Luria–Bertani (LB) Agar supplemented with 12.5 μg/ml chloramphenicol plate.

### Determination of average insert size of the constructed oral metagenomic library

To determine the average insert size of the constructed oral metagenomics library, 10 random colonies from the LB Agar chloramphenicol control plate were subcultured into 5 ml LB broth containing 12.5 μg/ml chloramphenicol and incubated at 37 °C with shaking at 200 RPM for 18 h. The plasmids containing the insert from each clone were extracted by using QIAprep Spin Miniprep Kit (QIAgen, Norway), following the manufacturer’s protocol. After extraction, each plasmid was digested in 10 μl reaction, containing 1 µl CutSmart buffer (10x), 0.5 μl HindIII restriction enzyme, 1 μl plasmid, and 7.5 μl distilled water. Each reaction was digested at 37 °C for 30 min. To visualize the inserts, each digested product was run on agarose gel electrophoresis with 120 V for one hour. The average insert size of the constructed library was calculated based on the insert size of each sample, estimated from the gel.

### Determination of minimum inhibitory concentration and screening of the oral metagenomic library

The minimum inhibitory concentration (MIC) of each antimicrobial was determined for *E. coli* BacRep containing pSMART BAC vector (with no insert), following the broth dilution method as described previously [38]. An overnight culture was set up by subculturing a single colony into 5 ml LB broth containing 12.5 μg/ml chloramphenicol and incubated for 18 h at 37 °C with shaking at 200 RPM. The overnight culture was diluted to the OD_600_ of 0.1. The MIC was determined in a 96-well microtiter plate by adding 10 μl of the diluted culture and 90 μl LB broth containing different concentrations of antimicrobial agents, shown in Table 1. The plates were incubated at 37 °C with shaking for 18–24 h, and the growth was determined by reading OD_600_ before and after incubation with a microplate spectrophotometer. This was repeated three times for each antimicrobial.

**Table 1:** Minimum inhibitory concentration (MIC) and the range of antimicrobials tested against *E. coli* BacRep containing empty pSMART BAC vector

Antimicrobial agents	MIC	Range
Sodium hypochlorite	0.025%	0.003125–0.1%
Chlorhexidine	1.0 μg/ml	0.313–10 μg/ml
Sodium benzoate	40 mg/ml	1.25–40 mg/ml
CTAB	4 μg/ml	1–32 μg/ml
Triclosan	30 μg/ml	0.16–80 μg/ml
Tetracycline	5 μg/ml	0.5–15 µg/ml
Erythromycin	175 μg/ml	75–200 μg/ml

For the screening of the oral metagenomic library for resistance clones, 100 μl of the electroporated *E. coli* carrying pSMART with oral metagenome insert were spread on LB agar supplemented with 12.5 μg/ml chloramphenicol and antimicrobial with the MIC concentration determined in the previous step, then incubated overnight at 37 °C. All of the colonies grown on the screening plates of each antimicrobial were streaked onto a new antimicrobial containing plate, and also subcultured into 5 ml LB broth containing the antimicrobial to confirm the resistance.

### Characterization of genes conferring antimicrobial resistance

The confirmed resistance clones were subcultured into 5 ml LB broth, containing chloramphenicol and antimicrobial of their resistance, and incubated at 37 °C for 18 h with shaking. The plasmids were extracted by using QIAprep Spin Miniprep Kit, digested with HindIII restriction enzyme, and visualized on an agarose gel to estimate the size of inserts.

All the inserts, except for the sodium hypochlorite-clone, were amplified by setting up PCR reactions with Platinum SuperFi Green PCR Master Mix (ThermoFisher Scientific, Norway), which can amplify up to 13 kb DNA, and SL1-SR4 primer pair (Lucigen, USA), which were the primers flanking the cloning site on the pSMART BAC vector. The 50-μl PCR reactions composed of 2 µl SL1 forward primer (10 μM), 2 µl SR4 reverse primer (10 μM), 25 µl 2 × Platinum SuperFi Green PCR Master Mix (Thermo Scientific, Norway), 20 µl molecular grade water, and 1 μl plasmid. The PCR cycle was programmed, as suggested by the manufacturer’s protocol. The PCR products were purified by using QIAquick PCR Purification Kit (Qiagen, Norway), then sent for Sanger sequencing from both ends with SL-1 and SR-4 primers at Genewiz, Germany. Additional primers were designed to extend the sequencing for samples that were not fully sequenced by the initial sequencing.

Sequencing data were aligned and manipulated by using BioEdit software version 7.2.0 (http://www.mbio.ncsu.edu/bioedit/bioedit.html). The contigs of each sample were assembled by using CAP3 contig assembly program [39]. The assembled sequences were compared with sequences in the nucleotide and protein databases by using BlastN and BlastX from the National Centre for Biotechnology Information (NCBI) [40]. The nucleotide sequences of all resistance clones were deposited in Genbank with the accession numbers MZ955857 to MZ955863.

### Subcloning of putative genes conferring chlorhexidine and sodium hypochlorite resistance

The putative resistance genes were amplified from the plasmids extracted from chlorhexidine and sodium hypochlorite resistant clones by using primers listed in Additional file 1: Table S1. The 30-μl PCR reactions composed of 15 µl 2 × BioMix Red (Bioline, United Kingdom), 2 μl of each primer (10 μM), 1 μl extracted plasmid and 10 μl molecular grade water. The PCR products were purified by using QIAquick PCR Purification Kit (Qiagen, Norway). All purified products, except *recA* PCR amplicons, were digested with HindIII and ligated to a HindIII-predigest pSMART BAC vector by using Anza T4 DNA Ligase Master Mix. The HindIII-ligated products were electroporated into BacRep *E. coli* Electrocompetent cells and grew on LB agar containing 12.5 μg/ml chloramphenicol. For the *recA* PCR amplicons, it was digested with EcoRI and ligated to an EcoRI-predigested pUC19 vector instead as there was an internal HindIII restriction site in the *recA* gene. The pUC19-*recA* ligation product was introduced into Subcloning Efficiency™ DH5α Competent Cells (Thermo Scientific, Norway) by heat-shock transformation and grew on LB agar containing 100 μg/ml ampicillin. The listed of bacterial strains and plasmids from the subcloning of the putative resistance genes were shown in Additional file 2: Table S2.

## Results

### Screening of the constructed oral metagenomic library against antimicrobials used in dental practice

After the construction of the metagenomic library, the average insert size was calculated by determining the insert size from 10 random colonies, which showed the average insert size of the constructed library as 5500 bp (Additional file 3: Fig. S1).

**Fig. 1:**
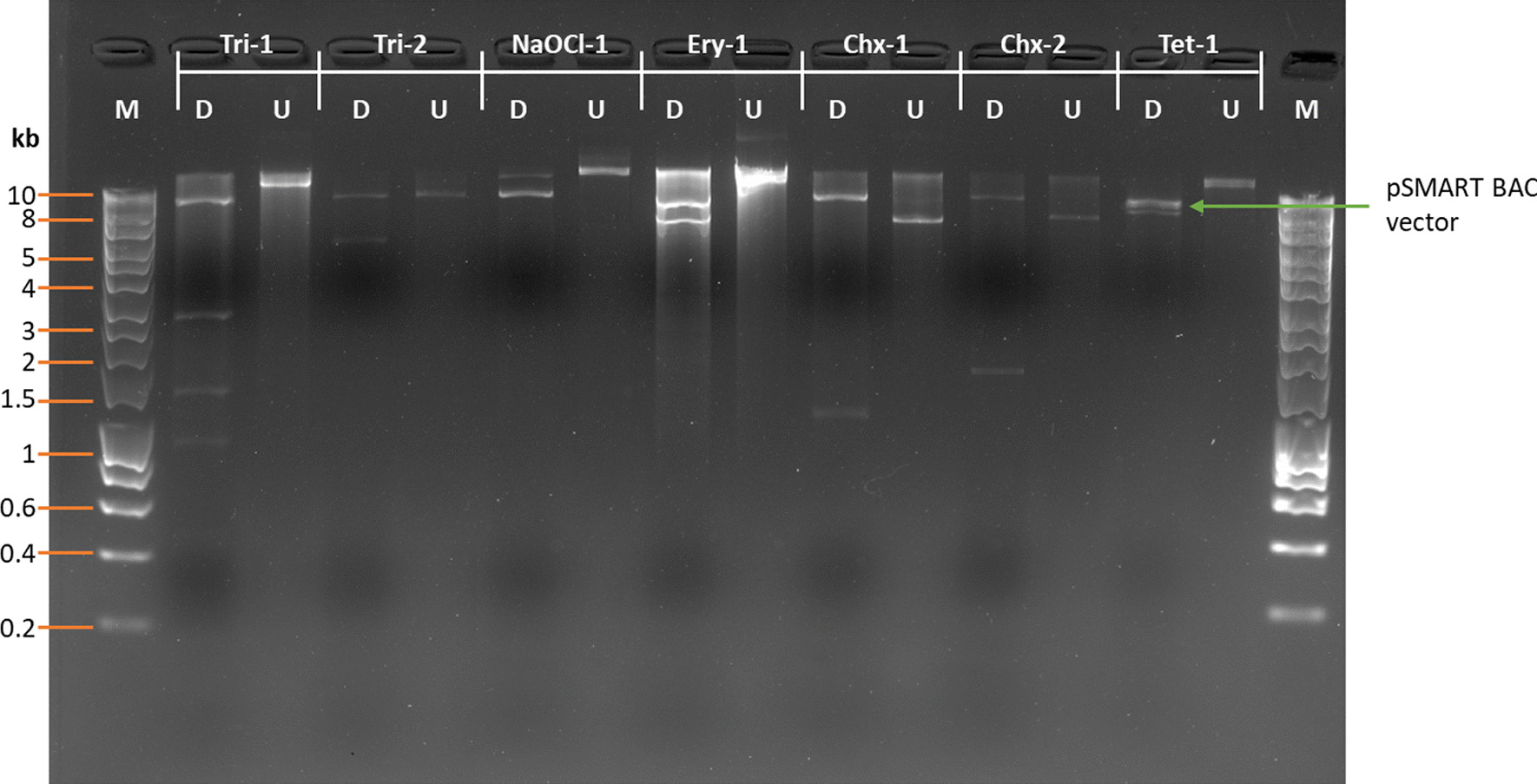
HindIII digestion of plasmids extracted from resistant clones identified from the oral metagenomic library. pSMART BAC vector backbone was indicated with the green arrow. Lane M, HyperLadder™ 1 kb. U, undigested plasmid; D, digested plasmid. The digested product was run on a GelRed® precast gel

**Fig. 2:**
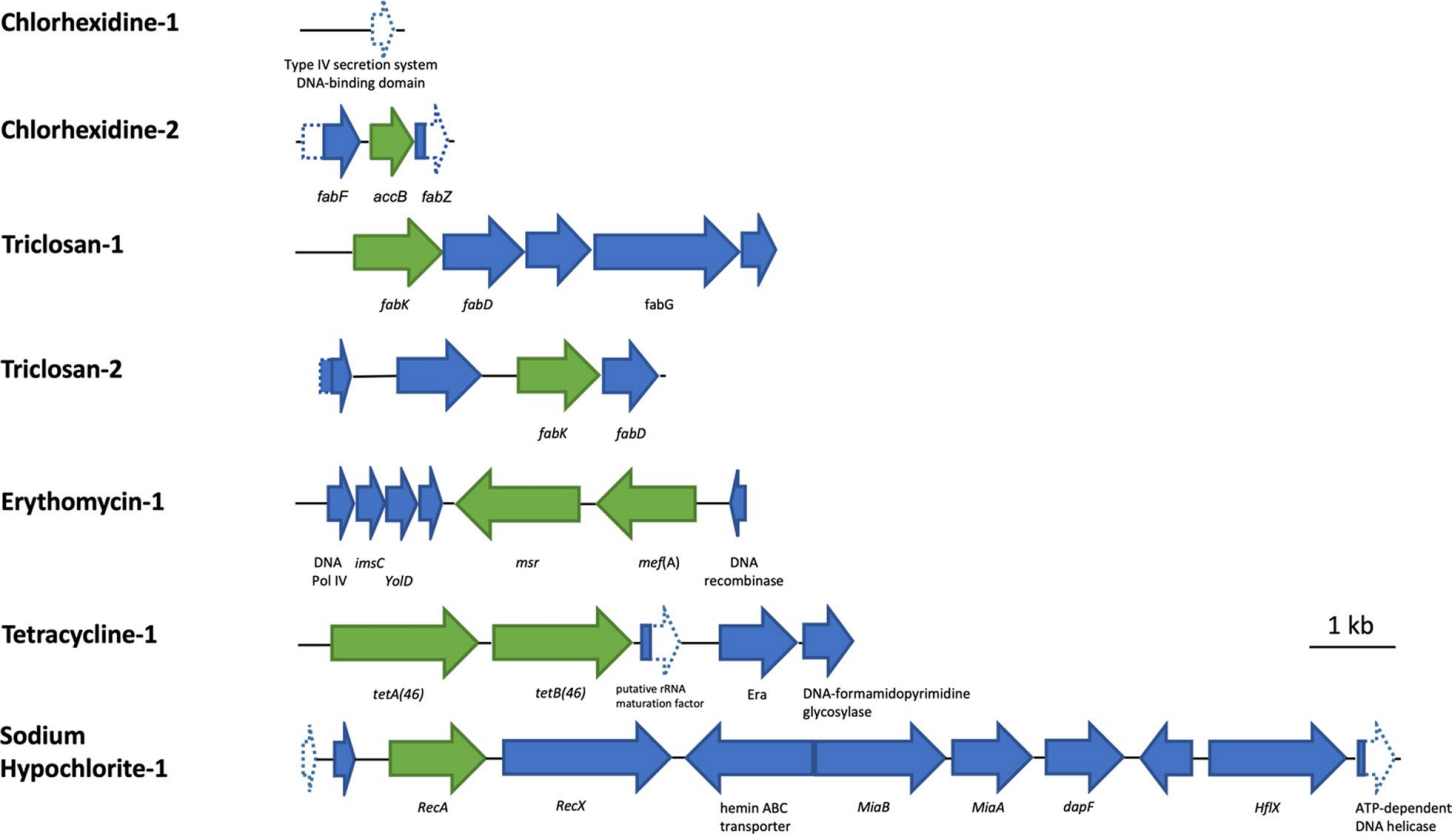
Schematic representation of predicted ORFs found on insert DNA of each resistant clone. The open arrowed boxes represent ORFs, pointing in the probable direction of transcription. The known resistant genes and other genes are shown in green and blue, respectively. The dash boxes and arrow boxes represent the regions that are not found on the inserts, compared to the sequences in the database

The MICs of *E. coli* BacRep carrying an empty pSMART BAC vector towards different antimicrobials were determined and listed in Table 1. The oral metagenomic library was then screened against each antimicrobial based on these MICs in which 7 different resistant clones were identified, including Chlorhexidine-1 (Chx-1), Chlorhexidine-2 (Chx-2), Triclosan-1 (Tric-1), Triclosan-2 (Tric-2), Erythromycin-1 (Ery-1), Tetracycline-1 (Tet-1) and Sodium hypochlorite-1 (NaOCl-1). HindIII plasmid digestion was performed to estimate the insert size of each resistant clone (Fig. 1). Sequencing and bioinformatics analysis through BlastN and BlastX of each clone were performed and shown in Fig. 2 and Table 2.

**Table 2:** Characterization of the DNA inserts from the resistant clones found in the oral metagenomic library

Sample name (Accession number)	Size (bp)	BlastN				BlastX					
		Closest homologue	Percentage identity (%)	Coverage (%)	Accession number of the homologous DNA (BlastN)	Closest homologue	ORF size (bp)	Percentage identity (%)	Coverage (%)	Position on sample	Accession number of the homologous proteins (BlastX)
Sodium hypochlorite- 1 (MZ955857)	12,588	*Rothia mucilaginosa* strain FDAARGOS_369 chromosome, complete genome	90.32	77	CP023510.1	Helix-turn-helix transcriptional regulator [*Rothia mucilaginosa*]	168	100	41	2 → 169	WP_049346502.1
						MULTISPECIES: DUF3046 domain-containing protein [*Rothia*]	222	100	100	347 → 568	WP_049333114.1
						MULTISPECIES: Recominase RecA [*Rothia*]	1107	99	100	979 → 2085	WP_049332756.1
						RecX family trancriptional regulator [*Rothia sp.* HMSC072E10]	1938	97	100	2292 → 4229	WP_070680682.1
						Hemin ABC transporter substrate-binding protein [*Rothia mucilaginosa*]	1476	98	100	4391 ← 5866	WP_152901671.1
						MULTISPECIES: tRNA (N6-isopentenyl adenosine(37)-C2)-methylthiotransferase MiaB [*Rothia*]	1515	100	100	5883 → 7397	WP_049332750.1
						tRNA (adenosine(37)-N6)-dimethylallyltransferase MiaA [*Rothia mucilaginosa*]	930	99	100	7474 → 8403	WP_193389320.1
						MULTISPECIES: Diaminopimelate epimerase [*Rothia*]	936	100	100	8501 → 9436	WP_049344416.1
						MULTISPECIES: Methyltransferase [*Rothia*]	624	100	100	9603 ← 10,226	WP_049332745.1
						GTPase HflX (*Rothia sp*. HMSC068F09)	1581	99	94.3	10,426 → 12,006	WP_070648960.1
						ATP-dependent DNA helicase [*Rothia sp.* HMSC072B03]	472	96	22.6	12,117 → 12,588	WP_070482790.1
Chlorhexidine- 1 (MZ955858)	1015	No significant similarity found	–	–	–	Type IV secretion system DNA-binding domain-containing protein [*Kocuria indica*]	237	51	10.8	746 → 982	WP_121544318.1
Chlorhexidine- 2 (MZ955859)	1536	*Streptococcus parasanguinis* ATCC 15,912, complete genome	99.28	92	CP002843.1	MULTISPECIES: Beta-ketoacyl-ACP synthase II [*Streptococcus*]	659	100	53.2	1 → 659	WP_014712692.1
						MULTISPECIES: Acetyl-CoA carboxylase biotin carboxyl carrier protein [*Streptococcus*]	489	100	100	663 → 1151	WP_061604604.1
						MULTISPECIES: 3-hydroxyacyl-ACP dehydratase FabZ [Bacteria]	267	99	62.4	1270 → 1536	WP_003004019.1
Erythromycin-1 (MZ955860)	5528	*Streptococcus mitis* strain SK637 chromosome, complete genome	99.74	96	CP028415.1	Hypothetical protein [*Streptococcus mitis*]	432	100	99.3	223 → 654	CDG57854.1
						MULTISPECIES: Hypothetical protein [Bacteria]	336	97	100	651 → 986	WP_000806926.1
						YolD-like protein [*Streptococcus pneumoniae*]	369	99	100	998 → 1366	VLH51388.1
						MULTISPECIES: Hypothetical protein [Bacteria]	300	100	100	1354 → 1654	WP_001072467.1
						ABC-F type ribosomal protection protein Msr(D) [*Streptococcus pneumoniae*]	2064	99	100	1771 ← 3234	WP_000420316.1
						Macrolide-efflux protein [*Streptococcus pneumoniae*]	1194	98	93	3379 ← 4572	VQI18798.1
						Hypothetical protein [*Streptococcus pneumoniae*]	186	100	100	4960 ← 5145	AAR22392.1
Triclosan-1 (MZ95561)	5408	*Streptococcus sp.* oral taxon 431, complete genome	91.83	99	CP014264.1	Enoyl-[acyl-carrier-protein] reductase FabK [*Streptococcus sp.* SK643]	1020	96	100	629 → 1648	WP_196793360.1
						ACP S-malonyltransferase [*Streptococcus sp.* HMSC034E03]	921	98	100	1641 → 2561	WP_075232029.1
						3-oxoacyl-[acyl-carrier-protein] reductase [*Streptococcus sp.* HMSC034E03]	735	99	100	2595 → 3329	WP_075232030.1
						Beta-ketoacyl-ACP synthase II [*Streptococcus mitis*]	1236	99	100	3352 → 4587	WP_075232031.1
						MULTISPECIES: 3-hydroxyacyl-ACP dehydratase FabZ [*Streptococcus*]	384	100	91.4	5025 → 5408	WP_042901453.1
Triclosan-2 (MZ955862)	3944	*Streptococcus salivarius* strain NCTC8618 genome assembly, chromosome: 1	99.75	97	LR134274.1	MULTISPECIES: Enoyl-CoA hydratase [*Streptococcus*]	389	100	49	1 → 389	WP_037601410.1
						MULTISPECIES: Ketoacyl-ACP synthase III [*Streptococcus*]	963	99	100	926 → 1888	WP_037612035.1
						MULTISPECIES: Enoyl-[acyl-carrier-protein] reductase FabK [*Streptococcus*]	966	100	100	2283 → 3248	WP_022495984.1
						ACP S-malonyltransferase [*Streptococcus salivarius*]	627	100	73.2	3318 → 3944	WP_049545679.1
Tetracycline-1 (MZ955863)	6366	*Streptococcus australis* strain NCTC13166 genome assembly, chromosome: 1	93.08	99	LS483444.1	Tetracycline efflux ABC transporter Tet(46) subunit A [*Streptococcus parasanguinis*]	1725	99	97.5	380 → 2104	WP_125826751.1
						Tetracycline efflux ABC transporter Tet(46) subunit B [*Streptococcus parasanguinis*]	1737	99	100	2106 → 3842	WP_049497012.1
						MULTISPECIES: rRNA maturation RNase YbeY [*Streptococcus*]	498	99	100	3939 → 4436	WP_003005431.1
						MULTISPECIES: GTPase Era [*Streptococcus*]	900	99	100	4845 → 5744	WP_003004993.1
						MULTISPECIES: DNA-formamidopyrimidine glycosylase [*Streptococcus*]	577	98	67	5790 → 6366	WP_070587080.1

### Identification of genes conferring resistance in the identified resistant clones

Among 7 resistant clones identified from the screening, 4 clones were shown to carry previously known resistance genes. For Ery-1, it contained *msr* and *mef(A)* macrolide resistance genes, that encode for a macrolide efflux pump [41, 42]. The tetracycline-resistant clone Tet-1 carried *tet*AB(46), an ABC-transporter which was isolated previously from the human oral cavity [24, 43]. It has been proved to transport tetracyclines over the cell membrane in both Gram-positive and Gram-negative bacteria, but as a single-drug efflux pump [43]. Both Tric-1 and Tric-2 contained *fabK* (an isoform of *fabI*), encoding for an enzyme, enoyl-acyl carrier protein reductase II (ENR). ENR is involved in the fatty acid synthesis, and also the binding site for triclosan to inhibit the synthesis. Upregulation of ENR resulted in a lower inhibitory effect from triclosan, as shown previously [1].

For chlorhexidine resistant clones (Chx-1 and Chx-2), bioinformatics analysis showed that they each contain only one complete open reading frame with the size of 750 bp and 489 bp, respectively. Blast analysis of the ORF in Chx-1 did not show any match by blastN and blastP. A partial match was shown by blastX that it had a 236-bp region that showed 51% similarity to a part of type IV secretion system DNA-binding domain-containing protein from *Kocuria indica*. The complete ORF in Chx-2 was matched to *accB* gene, encoding acetyl-CoA carboxylase biotin carboxyl carrier protein. As there were also another 2 incomplete ORFs in Chx-2, the *accB* gene of Chx-2 was amplified and subcloned into pSMART BAC vector to confirm that it is the only gene responsible for the chlorhexidine resistance phenotype. The MIC of chlorhexidine against *E. coli* BacRep::pSMART-Chx-1, *E. coli* BacRep::pSMART-Chx-2 and *E. coli* BacRep::pSMART-*accB* were shown to increase two-fold (from 1.0 to 2.0 µg/ml), compared to the wild-type *E. coli* BacRep::pSMART.

Bioinformatic analysis showed that the insert DNA of NaOCl-1 clone contained multiple genes, (as shown in Fig. 2 and Table 2) none of them was reported as a gene conferring sodium hypochlorite resistance. Four putative genes, encoding methyltransferase, diaminopimelate epimerase (DapF), hemin ABC transporter and RecA, were amplified and subcloned to determine the gene that conferred sodium hypochlorite resistance. The subcloning results showed that only *E. coli* DH5α::pUC19-*recA* showed an increase in MIC against sodium hypochlorite from 0.040 to 0.050%, compared to the wild-type *E. coli* DH5α::pUC19.

The distribution of all detected resistance genes was determined by performing BlastN on the metagenomic sequencing data of each saliva oral metagenomic DNA. The results showed that 6 of 7 detected resistance genes could be found in all 50 subjects, and the last one (Chx-1) could be found in 4 out of 50 subjects (Additional file 4).

## Discussion

Antimicrobial resistance is a major burden for the healthcare system worldwide. Several AMR genes have been discovered from the human oral microbiome previously. Some of them were found to be associated with mobile genetic elements, such as Tn*916* family conjugative transposons [44, 45], that can facilitate that spreading to other oral bacteria, including pathogens. As the oral cavity is the entry point to both the respiratory and gastrointestinal tract, oral bacteria that contain these resistance genes can therefore easily wander through the body via the bloodstream or by swallowing, and thereby have the potential to transfer their resistance genes to other microbiomes [25]. Therefore, it is important to screen and identify resistance genes in the oral microbiome so that we can design effective strategies and guidelines for antimicrobial uses to limit the spreading of these genes.

In our study, we used functional metagenomics to screen the oral metagenome for resistance genes against the antimicrobials used in dental practices, where 7 resistance clones were identified. Four of them contained previously known AMR, which also could be found in the oral cavity, where one of them (*fabI*) was found by functional metagenomic screening [1, 43, 46, 47]. The rest did not contain known resistance genes: two of them showed resistance to chlorhexidine, and another clone had resistance against sodium hypochlorite. This is the first time that functional metagenomic screening identified resistance genes for both antimicrobials from the oral metagenome.

Chlorhexidine is a widely used broad-spectrum antimicrobial in dentistry (e.g. endodontology, periodontology, oral surgery) [48]. It is normally combined with gluconid or acetic acid to form water-soluble digluconate or diacetate salts [49]. The mode of action is dose-dependent; bacteriostatic at low concentrations, bactericidal in higher, both through binding to negatively charged membrane phospholipids, resulting in reduced membrane fluidity and osmoregulation [50, 51]. Therefore, in lower concentrations, it disrupts the membrane causing leakage of low-weight molecules, while, in higher concentrations, it causes cytolysis by forming precipitates and releasing intracellular components [49, 50].

For the Chx-2 clone, the gene responsible for resistance was *accB*, encoding for biotin carboxyl carrier protein (BCCP) which is a component of acetyl CoA carboxylase that catalyzes the first step in fatty acid and phospholipid biosynthesis [52]. BCCP was previously reported to be one of the proteins that were upregulated as a consequence of chlorhexidine exposure in a proteomic analysis of a resistant *Pseudomonas aeruginosa* [53]. In our study, we showed and confirmed that *accB* conferred chlorhexidine resistance by expressing a heterologous *accB,* recovered from the oral cavity, in an *E. coli* surrogate host. Overexpressing *accB* in *E. coli* could increase the rate of phospholipid biosynthesis, which is the main component in the bacterial cell membrane, allowing the bacteria to become less sensitive against chlorhexidine which targets phospholipids in the cell membrane.

Sodium hypochlorite is an irrigation agent that is widely used in endodontic procedures, such as root canal fillings, by dentists. It is antimicrobial mainly in wet environments as it ionizes to Na^+^ and OCl^−^. At pH levels between 4 and 7, its form is hypochlorous acid (HClO), while at pH > 9 it is OCl^−^—both of them reactive oxidizing agents [54]. Teeth with pulp necrosis have a pH value between 6–7.4 before treatment, which means that HClO is the most important form of sodium hypochlorite for the treatments [55]. However, it is the tissue-dissolving property that may be considered the most important one for the procedures, where peptide bonds are destroyed, followed by dissolving proteins that can be irrigated away from the root canal [56].

The antimicrobial effect of sodium hypochlorite has been shown to be a complex process. As it is an oxidative antimicrobial, it causes oxidative stress which damage both DNA and lipids [57, 58]. We found that *recA*, isolated from the oral metagenome in our study, could confer sodium hypochlorite resistance when in *E. coli* host. RecA plays an important role in homologous recombination and DNA repairs like SOS response that is activated by DNA damage from various environmental factors and antibiotics. Previously, it was demonstrated that mutations in *recA* and *recB* repair genes increased the sensitivity an *E. coli* strains to HClO [59, 60]. In our case, as the *E. coli* lab strains (DH5α and BacRep) were *recA*-deficient strains, introducing pSMART BAC and pUC19 plasmids containing *recA* would result in complementation of *recA* in these *E. coli* lab strains, which was shown to have higher MIC against NaOCl.

It is a common practice in functional genomic studies to use *E. coli* to test the metagenomic constructed libraries. In the current study, *E. coli* was utilised to express *accB* and *recA* genes. Although the two genes are housekeeping genes, it is advisable to assess the expression of these genes in oral bacteria given their heterogeneous nature.

The two newly recognized AMR genes were not associated with mobile genetic elements, which implies that they are not able to be transferred or spread by themselves. However, it has been shown that selective pressure from uses of antimicrobials could drive these housekeeping genes to be associated with mobile genetic elements like *fabI* gene found in IS*1272* composite transposons [61], which give them the ability to be spread. Exposing bacteria to compounds like sodium hypochlorite could also lead to the spread of antibiotic resistance genes between bacteria, as it can induce an SOS-response in bacteria which promotes the spread of mobile genetic elements like integrative conjugative elements (SXT) that can facilitate an intercellular transposition [62].

Chlorhexidine is most used as a mouth rinse the days after a third mandibular molar surgery, to treat pericoronitis as well as it might be considered as an additional treatment for periodontitis patients with impaired access to adequate mechanical hygiene [63–65]. It is known that mouth rinses containing chlorhexidine reduce the amount of plaque in the oral cavity [21, 48], which is important as we know that the oral microbiota can play an important role in respiratory infections [66–68]. There is also a known relationship between the presence of oral bacteria, specifically viridans group streptococci, and infectious endocarditis (IE) [69–71]. A hot topic of discussion is the use of antibiotic prophylaxis in dental procedures that increase the risk of infectious endocarditis. In England, it has been observed that with more restrictive use of antibiotic prophylaxis, the incidence of IE has also increased [72]. Therefore, it is crucial for the health system to design a proper and well-balanced dental antimicrobial stewardship that can minimize the spread of AMR genes but still effectively prevent infection in dental treatments at the same time.

## Conclusion

In conclusion, multiple clones conferring resistance to antimicrobials commonly used in dental practices were detected in the studied population, proved to contain known and novel AMR genes when screened by functional-based metagenomics. This emphasizes the importance of reducing all use of antimicrobials, as this will reduce the selective pressure that could drive the spread of AMR genes even from commensal oral bacteria to pathogens. There is a need for more studies in this field to increase our knowledge regarding the AMR crisis.

## Supplementary Information

**Additional file 1**. Primers used in this study.

**Additional file 2**. Bacterial strains and plasmids used in the subcloning of putative chlorhexidine and sodium hypochlorite resistance genes.

**Additional file 3**. Estimation of the average insert size of the constructed human oral metagenomic library.

**Additional file 4**. BlastN results of the metagenomic sequencing data of each saliva oral metagenomic DNA against the resistance genes detected in this study.

## Data Availability

The datasets used and/or analysed during the current study available from the corresponding author on reasonable request.

## Abbreviations

AMR: Antimicrobial resistance
CTAB: Cetyltrimethylammonium bromide
BCCP: Biotin carboxyl carrier protein
Chx: Chlorhexidine
Tric: Triclosan
Ery: Erythromycin
Tet: Tetracycline
NaOCl: Sodium hypochlorite
MIC: Minimum inhibitory concentration
BacRep: BAC-optimized replicator
HClO: Hypochlorous acid
