# Supplementary material for: Functional screening of a human saliva metagenomic DNA reveal novel resistance genes against sodium hypochlorite and chlorhexidine
Source: BMC Oral Health. 2021 Dec 9;21:632. doi: 10.1186/s12903-021-02000-5 (PMC8656073; doi:10.1186/s12903-021-02000-5)
Supplement: Supplementary file 1 — Additional file 1. Primers used in this study. [file 12903_2021_2000_MOESM1_ESM.docx]

**Table S1.** Primers used in this study.

| **Primer name** | **Sequence (5’-3’)^a^** | **Gene target** | **Reference** |
| --- | --- | --- | --- |
| **Primer for initial sequencing from all resistant clones** | | | |
| SR-4 | TTTAGTATGGTTGTACCAGTT | End sequencing forward primer | Lucigen, USA |
| SL-1 | CAGTCCAGTTACGCTGGAGTC | End sequencing reverse primer | Lucigen, USA |
| **Sodium hypochlorite-1 (NaOCl-1)** | | | |
| NaOCl-1-F2 | AAAGGTGTACCCGATGCCGT | NaOCl-1 forward extension | This study |
| NaOCl-1-F3 | CGGCCTCAGATACGGAATTA | NaOCl-1 forward extension | This study |
| NaOCl-1-R2 | CGTGGCTAAGAGCACCAAGT | NaOCl-1 reverse extension | This study |
| NaOCl-1-R3 | CGTAAGCTTCTCAAGGACAA | NaOCl-1 reverse extension | This study |
| NaOCl-1-R4 | TGCGCAGGAAAACGTAACC | NaOCl-1 reverse extension | This study |
| NaOCl-1-R5 | GCCTTTATTCGGGGTATCGC | NaOCl-1 reverse extension | This study |
| NaOCl-1-R6 | ACCCTATAAAGCCCCTGTCG | NaOCl-1 reverse extension | This study |
| NaOCl-1-R7 | GCTCATCCACGCCGTAAATT | NaOCl-1 reverse extension | This study |
| NaOCl-1-R8 | GGCATGACTTCGACCCTCA | NaOCl-1 reverse extension | This study |
| NaOCl-1-R9 | GTGAAATTGAGGGCCTGGAG | NaOCl-1 reverse extension | This study |
| NaOCl-1-R10 | CGAAACTCTGCCCGTTATCG | NaOCl-1 reverse extension | This study |
| **Chlorhexidine-1 (Chx-1)** | | | |
| Chx-1-F1 | TGGTCTGCTGGCTATCTCTG | Chx-1 forward extension | This study |
| Chx-1-R1 | CACCAACCACACACACTACG | Chx-1 reverse extension | This study |
| **Chlorhexidine-2 (Chx-2)** | | | |
| Chx-2-F1 | CAAGGCCTCACGAATAGCAT | Chx-2 forward extension | This study |
| Chx-2-R2 | ACACCTGCCAATGAGAAAGG | Chx-2 reverse extension | This study |
| **Erythromycin-1 (Ery-1)** | | | |
| Ery-1-F1 | GGAACCGAAACTATGACAGCC | Ery-1 forward extension | This study |
| Ery-1-F2 | CACTTGCTATGCCAATTGGG | Ery-1 forward extension | This study |
| Ery-1-R1 | GGAACCGAAACTATGACAGCC | Ery-1 reverse extension | This study |
| Ery-1-R2 | GGAAGTTACTGGGTTCATCC | Ery-1 reverse extension | This study |
| **Triclosan-1 (Tri-1)** | | | |
| Tri-1-F2 | CTGGATTTCTGCCCGCATTAT | Tri-1 forward extension | This study |
| Tri-1-R2 | GTACCTTGGAATGGGACGTG | Tri-1 reverse extension | This study |
| Tri-1-R3 | GGTTCAAGTCGAGGAATTGG | Tri-1 reverse extension | This study |
| **Triclosan-2 (Tri-2)** | | | |
| Tri-2-F2 | GCAACAAGGTCACCAACAGT | Tri-2 forward extension | This study |
| Tri-2-F3 | GTCAAGGGGCTCAGAAATTG | Tri-2 forward extension | This study |
| **Tetracycline-1 (Tet-1)** | | | |
| Tet-1-F2 | CCTTGAAGGGCTGAAATCGG | Tet-1 forward extension | This study |
| Tet-1-F3 | CATATTCTTCGGCTTGCTCAC | Tet-1 forward extension | This study |
| Tet-1-F4 | ACTGGATGGTCGATTCCCTT | Tet-1 forward extension | This study |
| Tet-1-F5 | ATCCCCATATGGCTGCGTAA | Tet-1 forward extension | This study |
| Tet-1-R2 | AGCAGGTCATACAGTTTCAG | Tet-1 reverse extension | This study |
| Tet-1-R3 | GAAGAAGGCAGGGCTAGTGA | Tet-1 reverse extension | This study |
| Tet-1-R4 | GGGAAGATCGCGACAAGAAT | Tet-1 reverse extension | This study |
| Tet-1-R5 | CAATATCGGTGTGACGGCTG | Tet-1 reverse extension | This study |
| **Primers for subcloning of putative resistance genes** | | | |
| dapF-F | CCGGCG**AAGCTT** GCTTCCGTATTCTGCAGGTG | *dapF* forward primer | This study |
| dapF-R | GCGGCG**AAGCTT** GAAAGCACCCGATAAGCCAC | *dapF* reverse primer | This study |
| Hemin-F | GCCGGG**AAGCTT** CAATCTGCATACCCTCATGG | Hemin forward primer | This study |
| Hemin-R | CCCGGG**AAGCTT** CGGCTACTCCTCCTCGGTAT | Hemin reverse primer | This study |
| Methyl-F | GCCGGG**AAGCTT** TCGTAGGTGGTCAGTGAGGA | Methyltransferase forward primer | This study |
| Methyl-R | CCCGGG**AAGCTT** CTAAGGTACTGCCCCGGAAC | Methyltransferase reverse primer | This study |
| RecA-F | CCGGGG**GAATTC** GTCCTCGCGTATACCGTCAC | *recA* forward primer | This study |
| RecA-R | CGGGCG**GAATTC** CTGGGCGGTGTGTCTAAATG | *recA* reverse primer | This study |
| accB-F1 | CGCGGG**AAGCTT** GCGATTCGTCACAGCTTTGT | *accB* forward primer | This study |
| accB-R1 | GCGGCG**AAGCTT** CCATGATGAGAACACCTGGC | *accB* reverse primer | This study |

^a^ Restriction sites were indicated as bold style
