# Supplementary material for: Functional screening of a human saliva metagenomic DNA reveal novel resistance genes against sodium hypochlorite and chlorhexidine
Source: BMC Oral Health. 2021 Dec 9;21:632. doi: 10.1186/s12903-021-02000-5 (PMC8656073; doi:10.1186/s12903-021-02000-5)
Supplement: Supplementary file 2 — Additional file 2. Bacterial strains and plasmids used in the subcloning of putative chlorhexidine and sodium hypochlorite resistance genes. [file 12903_2021_2000_MOESM2_ESM.docx]

**Table S2.** Bacterial strains and plasmids used in the subcloning of putative chlorhexidine and sodium hypochlorite resistance genes.

| **Strains or Plasmids** | **Characteristics** | **Resistance phenotypes** | **References** |
| --- | --- | --- | --- |
| **Strains** |  |  |  |
| *E. coli* |  |  |  |
| BAC-Optimized   Replicator | - Electrocompetent cells | Ampicilin | Lucigen, USA |
| BacRep::pSMART | - *E. coli* BAC-Optimized Replicator containing empty pSMART BAC HindIII vector | Ampicillin, Chloramphenicol | This study |
| BacRep::pSMART-Chx-1 | - *E. coli* BAC-Optimized Replicator containing pSMART-Chx-1 | Ampicillin, Chloramphenicol, Chlorhexidine | This study |
| BacRep::pSMART-Chx-2 | - *E. coli* BAC-Optimized Replicator containing pSMART-Chx-2 | Ampicillin, Chloramphenicol, Chlorhexidine | This study |
| BacRep::pSMART-*accB* | - *E. coli* BAC-Optimized Replicator containing pSMART-*accB* | Ampicillin, Chloramphenicol, Chlorhexidine | This study |
| BacRep::pSMART-NaOCl-1 | - *E. coli* BAC-Optimized Replicator containing pSMART-NaOCl-1 | Ampicillin, Chloramphenicol,  Sodium hypochlorite | This study |
| BacRep::pSMART-*dapF* | - *E. coli* BAC-Optimized Replicator containing pSMART-*dapF* | Ampicillin, Chloramphenicol | This study |
| BacRep::pSMART-methyl | - *E. coli* BAC-Optimized Replicator containing pSMART-methyl | Ampicillin, Chloramphenicol | This study |
| BacRep::pSMART-hemin | - *E. coli* BAC-Optimized Replicator containing pSMART-hemin | Ampicillin, Chloramphenicol | This study |
| Subcloning Efficiency™   DH5α | - Chemically competent cells | - | Thermo Fisher Scientific, Norway |
| DH5α::pUC19 | - *E. coli* Subcloning Efficiency™   DH5α containing empty pUC19 vector | Ampicillin | This study |
| DH5α::pUC19-*recA* | -*E. coli* Subcloning Efficiency™   DH5α containing pUC19-*recA* | Ampicillin, Sodium hypochlorite | This study |
| **Plasmids** |  |  |  |
| pSMART BAC HindIII | -Large insert, Single copy but inducible in *E. coli* BAC-Optimized Replicator | Chloramphenicol | Lucigen, USA |
| pSMART-Chx-1 | - pSMART BAC HindIII containing Chx-1 insert DNA | Chloramphenicol, Chlohexidine | This study |
| pSMART-Chx-2 | - pSMART BAC HindIII containing Chx-2 insert DNA | Chloramphenicol, Chlohexidine | This study |
| pSMART-*accB* | - pSMART BAC HindIII containing *accB* gene | Chloramphenicol, Chlohexidine | This study |
| pSMART-NaOCl-1 | - pSMART BAC HindIII containing NaOCl-1 insert DNA | Chloramphenicol, Sodium hypochlorite | This study |
| pSMART-*dapF* | - pSMART BAC HindIII containing *dapF* gene | Chloramphenicol | This study |
| pSMART-Methyl | - pSMART BAC HindIII containing methyltransferase gene | Chloramphenicol | This study |
| pSMART-Hemin | - pSMART BAC HindIII containing hemin ABC transporter gene | Chloramphenicol | This study |
| pUC19 | -Small insert, High copy number cloning vector | Ampicillin | New England Biolabs, UK |
| pUC19-*recA* | -pUC19 containing *recA* gene | Ampicillin, Sodium hypochlorite | This study |
