# Supplementary material for: Functional screening of a human saliva metagenomic DNA reveal novel resistance genes against sodium hypochlorite and chlorhexidine
Source: BMC Oral Health. 2021 Dec 9;21:632. doi: 10.1186/s12903-021-02000-5 (PMC8656073; doi:10.1186/s12903-021-02000-5)
Supplement: Supplementary file 3 — Additional file 3. Estimation of the average insert size of the constructed human oral metagenomic library. [file 12903_2021_2000_MOESM3_ESM.docx]

**
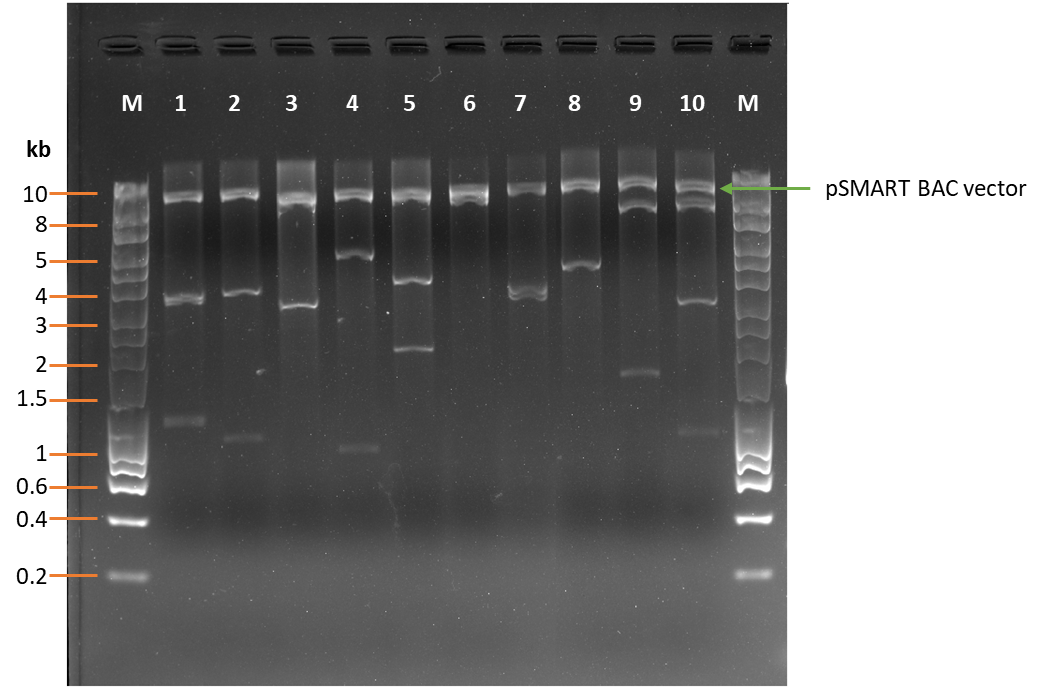
**

**Figure S1. Estimation of the average insert size of the constructed human oral metagenomic library.** pSMART BAC vector backbone was indicated with the green arrow. Lane M, HyperLadder™ 1kb. Lane 1-10 contain HindIII digested pSMART::insert vectors from 10 randomly selected clones from the human saliva metagenomic library. The digested product was run on a GelRed® precast gel.
